# Myocardial protection with phosphocreatine in high-risk cardiac surgery patients: a randomized trial

**DOI:** 10.1186/s12871-023-02341-4

**Published:** 2023-11-29

**Authors:** Vladimir Lomivorotov, Dmitry Merekin, Evgeny Fominskiy, Dmitry Ponomarev, Alexander Bogachev-Prokophiev, Anton Zalesov, Alexander Cherniavsky, Anna Shilova, Dmitry Guvakov, Liudmila Lomivorotova, Rosalba Lembo, Giovanni Landoni

**Affiliations:** 1 Department of Anesthesiology and Perioperative Medicine, Penn State College of Medicine, Penn State Milton S. Hershey Medical Center, Hershey, PA USA; 2 Department of Anesthesiology and Intensive Care, E. Meshalkin National Medical Research Center, Novosibirsk, Russia; 3 Department of Anesthesia and Intensive Care, IRCCS San Raffaele Scientific Institute, Via Olgettina 60, 20132 Milan, Italy; 4 Department of Heart Valve Surgery, E. Meshalkin National Medical Research Center, Novosibirsk, Russia; 5 Department of Aortic and Coronary Artery Surgery, E. Meshalkin National Medical Research Center, Novosibirsk, Russia; 6 Vita-Salute San Raffaele University, Milan, Italy

**Keywords:** Anesthesia, Intensive care, Phosphocreatine, Cardiopulmonary bypass, Valve heart surgery, Troponin, Myocardial protection, Cardioplegia

## Abstract

**Background:**

This study was conducted to test the hypothesis that phosphocreatine (PCr), administered intravenously and as cardioplegia adjuvant in patients undergoing cardiac surgery with prolonged aortic cross clamping and cardiopulmonary bypass (CPB) time, would decrease troponin I concentration after surgery.

**Methods:**

In this randomized, double-blind, placebo-controlled pilot study we included 120 patients undergoing double/triple valve repair/replacement under cardiopulmonary bypass in the cardiac surgery department of a tertiary hospital.

The treatment group received: intravenous administration of 2 g of PCr after anesthesia induction; 2.5 g of PCr in every 1 L of cardioplegic solution (concentration = 10 mmol/L); intravenous administration of 2 g of PCr immediately after heart recovery following aorta declamping; 4 g of PCr at intensive care unit admission. The control group received an equivolume dose of normosaline.

**Results:**

The primary endpoint was peak concentration of troponin I after surgery. Secondary endpoints included peak concentration of serum creatinine, need for, and dosage of inotropic support, number of defibrillations after aortic declamping, incidence of arrhythmias, duration of Intensive Care Unit (ICU) stay, length of hospitalization. There was no difference in peak troponin I concentration after surgery (PCr, 10,508 pg/ml [IQR 6,838–19,034]; placebo, 11,328 pg/ml [IQR 7.660–22.894]; *p* = 0.24). There were also no differences in median peak serum creatinine (PCr, 100 µmol/L [IQR 85.0–117.0]; placebo, 99.5 µmol/L [IQR 90.0–117.0]; *p* = 0.87), the number of patients on vasopressor/inotropic agents (PCr, 49 [88%]; placebo, 57 [91%]; *p* = 0.60), the inotropic score on postoperative day 1 (PCr, 4.0 (0–7); placebo, 4.0 (0–10); *p* = 0.47), mean SOFA score on postoperative day 1 (PCr, 5.25 ± 2.33; placebo, 5,45 ± 2,65; *p* = 0.83), need for defibrillation after declamping of aorta (PCr, 22 [39%]; placebo, 25 [40%]; *p* = 0.9),, duration of ICU stay and length of hospitalization as well as 30-day mortality (PCr, 0 (0%); placebo,1 (4.3%); *p* = 0.4).

**Conclusion:**

PCr administration to patients undergoing double/triple valve surgery under cardiopulmonary bypass is safe but is not associated with a decrease in troponin I concentration. Phosphocreatine had no beneficial effect on clinical outcomes after surgery.

**Trial registration:**

The study is registered at ClinicalTrials.gov with the Identifier: NCT02757443. First posted (published): 02/05/2016.

## Background

Perioperative myocardial injury is a common and serious complication in adult patients undergoing cardiac surgery under cardiopulmonary bypass (CPB) [1]. The extent of troponin release in cardiac surgery closely correlates with unfavorable outcomes, especially short- and mid-term mortality [1, 2].

Several factors may contribute to elevation of troponin in patients undergoing cardiac surgery under CPB. Inadequate myocardial protection coupled with surgical manipulations, reperfusion injury and oxygen/supply disbalance promote myocardial ischemia and lead to myocardial injury and increased troponin release. Therefore, adequate myocardial protection is the cornerstone in the management of patients undergoing cardiac surgery under cardiopulmonary bypass.

Modulation of substrate utilization and increasing myocardial high-energy phosphate stores seems to be a promising strategy to reduce the sequelae of myocardial injury. The phosphocreatine system plays an important role in the energy metabolism of cardiomyocytes. According to the “shuttle” hypothesis the main goal of this system is to provide transport of high-energy phosphates from the sites of adenosine triphosphate (ATP) production (mitochondrial matrix) to the sites of ATP utilization (myofilaments, sarcoplasmic reticulum, plasma membrane, etc.) [3].

Numerous clinical studies suggested favorable effects of phosphocreatine (PCr) supplementation in different patient populations including congestive heart failure, cardiac surgery, myocardial infarction and cerebral ischemia [4]. In a meta-analysis of randomized and matched studies, Landoni et al. showed a reduction in all-cause mortality in patients with coronary artery disease, chronic heart failure or in those undergoing cardiac surgery with PCr supplementation [5]. A recent meta-analysis of randomized trials suggested that treatment with PCr reduces troponin release in cardiac surgical patients [6]. Nevertheless, the major limitation of these meta-analyses was the inclusion of studies with high risk of bias and the heterogenous patient populations.

As above described, there is extraordinary experimental evidence on the essential role of the PCr system in the energy metabolism of the heart, with altered energetics playing an important role in the mechanisms of heart failure. At the same time there is a plenty of low-quality clinical evidence that accumulated over the years in this field suggesting that PCr and its analogues have cardioprotective qualities that can translate into improved clinically relevant outcomes.

We hypothesized that perioperative PCr intravenous administration plus PCr added to the cardioplegic solution in patients with double/triple valve procedures undergoing cardiac surgery with prolonged periods of aortic cross clamping and CPB would improve heart protection in postoperative period. We also aimed to identify the best endpoint for a future adequately powered study.

## Methods

The PRISE (myocardial protection with Phosphocreatine in high-RIsk cardiac SurgEry patients) study was approved by the local ethical committee and written informed consent was obtained from all patients before inclusion to the study (ClinicalTrials.gov Identifier: NCT02757443). One hundred and twenty patients aged 18 years and older scheduled for double/triple valve repair/replacement with CPB were enrolled. Exclusion criteria were emergency surgery, concomitant coronary artery bypass grafting surgery or procedure on any part of the aorta, concomitant radiofrequency/cryo- ablation procedure, concomitant left ventricular outflow tract myomectomy (Morrow procedure), chronic kidney disease of G3 and lower categories according to Kidney Disease: Improving Global Outcomes (KDIGO) criteria (at least one of the following present for > 3 months: glomerular filtration rate ≤ 60 ml/min/1.73 m^2^, history of kidney transplantation) or solitary kidney (by any reason), structural abnormalities or genetic trait point to kidney disease including glomerulonephritis and gout, known allergy to phosphocreatine, pregnancy, enrollment into another randomized controlled study in the last 30 days, previous enrollment and randomization into the PRISE trial, administration of phosphocreatine in the previous 30 day.

### Interventions

The treatment group received: intravenous administration of 2 g of PCr after anesthesia induction; 2.5 g of PCr in every 1 L of cardioplegic solution (concentration = 10 mmol/L); intravenous administration of 2 g of PCr immediately after heart recovery following aorta declamping; 4 g of PCr at intensive care unit admission. To ensure biochemical and clinical effects of the drug, PCr was administered perioperatively at a dose of 13 g (this dose was higher than previously reported in clinical trials) [5]. The price of one vial (1 g) of PCr is 13 US dollars. Therefore, the price of 13 g of PCr per one patient randomized to treatment group was 171 US dollars. The control group received an equivolume dose of normosaline.

The randomization was accomplished using an online randomization service (http://www.sealedenvelope.com). Allocation concealment was maintained using sequentially numbered, sealed, opaque envelopes containing the numerical code of the study arm to which the patient was randomized. Randomization was performed on the morning of surgery. Patients, anesthesiologists, intensive care specialists, and statistician, were blinded to patient allocation.

### Anesthetic management

Anesthesia induction and maintenance were similar for all patients and consisted of sevoflurane, fentanyl, rocuronium, and propofol. Cefuroxime was used perioperatively for surgical site infection prophylaxis. Volume-controlled ventilation intraoperatively and after surgery was provided at a fraction of inspired oxygen of 0.5, a tidal volume of 6 to 8 mL/kg, a respiratory rate of 12 to 14 breaths/min, and positive end-expiratory pressure of 5 cmH_2_O (Perseus a 500, Primus, Evita XL; Drager, Germany). The radial artery was catheterized using a 20G catheter (B Braun, Melsungen, Germany). A pulmonary artery catheter (7.5 Fr, five-lumen, Corodyn TDI, B Braun) and a triple-lumen central venous catheter (Certofix, B Braun) were inserted via the right internal jugular vein.

Nonpulsatile CPB mode was used with a perfusion index of 2.4 to 2.8 L/min/m^2^. Conventional median sternotomy was used in all patients. For CPB, standard cannulation of the ascending aorta, and either right atrium or upper and lower vena cava, was performed. Before connection to the extracorporeal circuit, patients were heparinized using 3 mg/kg of sodium heparin, maintaining an activated coagulation time of > 480 s during CPB. The circuit was primed with 1,000 mL of balanced crystalloid solution, 200 mL of 15% mannitol, and 150 mL of 5% sodium hydrocarbonate. Continuous infusion of aminocapronic acid (20 g) starting after anesthesia induction was used in all patients.

Mean arterial pressure (MAP) during CPB was maintained between 70 and 80 mmHg by administration of phenylephrine when necessary. For myocardial protection, 2,000 mL (single dose) of ice-cold (4 °C-6 °C) cardioplegic solution (Custodiol; HTK-Bretschneider, Dr. Franz Kohler Chemie GmbH, Bensheim, Germany) was delivered antegradely. At the termination of CPB, heparin was reversed using protamine sulfate at a ratio of 1:1. Steroids and aprotinin were not used. Packed red blood cells were administered when hemoglobin level dropped to < 8 g/dL. Fresh frozen plasma was transfused in cases of ongoing bleeding (150 mL/h blood loss over a 2-h period) based on abnormal coagulation parameters.

Inotropic support (epinephrine, dobutamine) was guided by hemodynamic data (eg, cardiac index < 2.0 L/min/m^2^ in the presence of a pulmonary capillary wedge pressure > 15 mmHg) as well as the institution of vasopressor support (norepinephrine: MAP < 65 mmHg despite a cardiac index > 2.2 L/min/m^2^ and a pulmonary capillary wedge pressure > 15 mmHg). Cardiac output was determined using the thermodilution method. Troponin I concentration was measured at baseline, after ICU admission, and on postoperative day 1, postoperative day 2, and postoperative day 3. Cardiac index was evaluated after anesthesia induction, 5 min after CPB, 6 h after Intensive Care Unit (ICU) admission and on the morning of postoperative day 1.

After surgery, all patients were transferred to the ICU. Patients were transferred from the ICU to the ward after meeting the following criteria: fully oriented; arterial oxygen saturation > 90% on room air; no episodes of severe arrhythmia; absence of bleeding; diuresis > 0.5 mL/kg/h; no need for inotropes and/or vasopressors; and no signs of myocardial ischemia on electrocardiography.

Patients were discharged from hospital when they met the following criteria: hemodynamic stability; independent ambulation and feeding; afebrile with no obvious infections; normal voiding and bowel function; pain control on oral medication(s); and able to tolerate exercise.

### Outcomes

The primary outcomes of the study was peak troponin I concentration after cardiac surgery. Secondary endpoints were: peak serum creatinine, number of patients receiving vasopressor/inotropic agents, the inotropic score at the end of surgery [7] and on postoperative day 1, mean Sequential Organ Failure Assessment (SOFA) score, need for defibrillation after declamping aorta, cardiac index, the incidence of acute kidney injury (AKI) according to Kidney Disease Improving Global Outcomes (KDIGO) criteria [8], rate of atrial fibrillation, duration of ventilation, duration of intensive care unit stay, length of hospitalization, 30-day mortality (within 30 days if at home; any time if still in hospital).

### Statistical analysis

Categorical data are presented as absolutes number and percentages. Categorical data were compared by two tailed χ^2^ test or Fisher’s exact test when appropriate. Continuous data were presented as median and interquartile ranges (IQR – 25^th^ and 75^th^ percentiles) or as mean and Standard Deviation (SD). Means and SD were used when the variable was normally distributed while medians and IQR were used with non-normally distributed variables. Normality was verified with Skewness and Kurtosis tests. Continuous measurements were compared using the Wilcoxon (Mann–Whitney) test or T test of Student if data were normally distributed. Two-sided significance test is used throughout. Anova was calculated for the Cardiac Index repeated values. All statistical analyses were performed with the STATA software (ver. 16; Texas USA).

Due to the pilot design of the study, we used a convenience sample-size of 120 patients. One of the reasons of doing this study was to identify the best endpoint for a future adequately powered study.

## Results

**Fig. 1:**
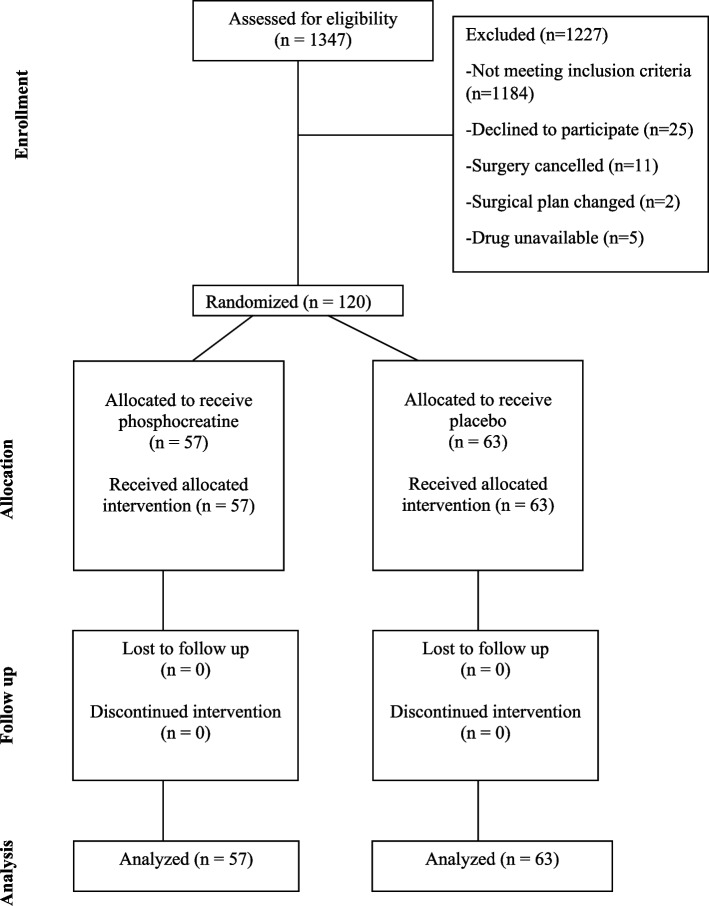
Flow-chart illustrating patient recruitment and randomization

From September 2016 to April 2021, 120 patients scheduled for double/triple valve repair/replacement were randomly assigned to receive either phosphocreatine or placebo (Fig. 1). All patients received assigned intervention. None of the patients withdrawn the consent to participate in the study.

Groups were well balanced at baseline (Table 1) and as intraoperative characteristics (Table 2). The median age was 57 years, left ventricular ejection fraction was 64% with an Euroscore II score of 2.0. The mean duration of CPB and aortic cross-clamping was 104 and 77 min, respectively. The most commonly performed procedures were mitral valve replacement (64%) and tricuspid valve repair (67%).

**Table 1:** Baseline demographic and clinical characteristics

	Placebo (*n* = 63)	Phosphocreatine (*n* = 57)
Age (y)	57 (46–65)	58 (51–64)
LVEF (%)	64 (58–70)	63 (57–66)
EUROSCORE II	1.88 (1.28–2.99)	2.2 (1.6–2.99)
Males %	29 (46%)	18 (31%)
Redo cardiac surgery	8 (13%)	6 (11%)
Previous MI	1 (1.6%)	0
Hypertension	21 (33%)	22 (39%)
Atrial fibrillation	20 (32%)	27 (47%)
COPD	5 (7.9%)	3 (5.3%)
Carotid stenosis	4 (6.3%)	4 (7.0%)
Previous stroke/TIA	6 (9.5%)	2 (2.5%)
Diabetes mellitus	5 (7.9%)	5 (8.8%)
Active smokers	4 (6.4%)	4 (7.0%)
Cardiac catheterization < 48 h of surgery	16 (26%)	11 (19%)
Medications		
Diuretics	30 (48%)	36 (63%)
Beta-blockers	22 (36%)	28 (49%)
ACE inhibitors	14 (23%)	16 (28)
Anticoagulants	15 (24%)	15 (26%)
Statins	9 (15%)	5 (8.8%)
Digoxin	5 (7.9%)	7 (12%)
Platelet inhibitors	4 (6.4%)	4 (7.0%)
ARBs	4 (6.4%)	7 (12%)
Calcium channel blockers	4 (6.4%)	3 (5.3%)
Ivabradine	1 (1.6%)	1 (1.7%)
NYHA class		
I	4 (6.4%)	1 (1.8%)
II	23 (85%)	20 (95%)
III	35 (56%)	34 (61%)
IV	1 (1.6%)	1 (1.8%)

Median (25^th^-75^th^ percentiles), or number of patients (percentage)

*Abbreviations*: *LVEF* Left ventricular ejection fraction, *EuroSCORE* European System for Cardiac Operative Risk Evaluation, *MI* Myocardial infarction, *COPD* Chronic obstructive pulmonary disease, *TIA* Transient ischemic attack, *ARBs* Angiotensin II receptor blockers, *ACE* Angiotensin-converting enzyme, *NYHA* New York Heart Association Classification

**Table 2:** Intraoperative characteristics

	Placebo (*n* = 63)	Phosphocreatine (*n* = 57)	*p*-value
Cardiopulmonary bypass time, minutes – median (IQR)	110 (88-133)	99 (87-118)	0.28
Aortic cross-clamp time, minutes – median (IQR)	79 (62-99)	75 (62-90)	0.41
Volume of cardioplegia, ml – median (IQR)	2100 (2100-2100)	2100 (2100-2100)	0.34
Mitral valve surgery			
repair, n (%)	20 (33%)	20 (36%)	0.74
replacement, n (%)	41 (65%)	36 (63%)	0.83
Aortic valve surgery			
repair, n (%)	5 (7.9%)	2 (3.5%)	0.30
replacement, n (%)	24 (38%)	19 (33%)	0.60
Tricuspid valve surgery			
repair, n (%)	42 (67%)	39 (68%)	0.84
replacement, n (%)	4 (6.3%)	4 (7.0%)	0.88
Other additional surgeries, n (%)	16 (25%)	14 (25%)	0.90
Need for defibrillation after aorta declamping, n (%)	25 (40%)	22 (39%)	0.90
Ventricular fibrillation while still in theatre after protamine administration, n	1 (1.6%)	1 (1.7%)	0.94
Ventricular tachycardia while still in theatre after protamine administration, n	1 (1.6%)	0	0.34
AV block grade 1, n (%)	0	1 (1.8%)	0.29
AV block grade 2, n (%)	2 (3.2%)	1 (1.8%)	0.62
AV block grade 3, n (%)	9 (14%)	7 (12%)	0.75
Inotropic score at the end of surgery – median (IQR)	5 (3-13)	5 (2-9)	0.47

NOTE. Data are presented as Median and IQR(Inter Quartile Range) (25^th^-75^th^ percentiles), or number of patients (percentage)

*Abbreviations*: *AV* atrio-ventricular

After surgery, patients were mechanically ventilated for a mean of 6 h and stayed in ICU for a mean of 17 h without differences between two groups.

**Fig. 2:**
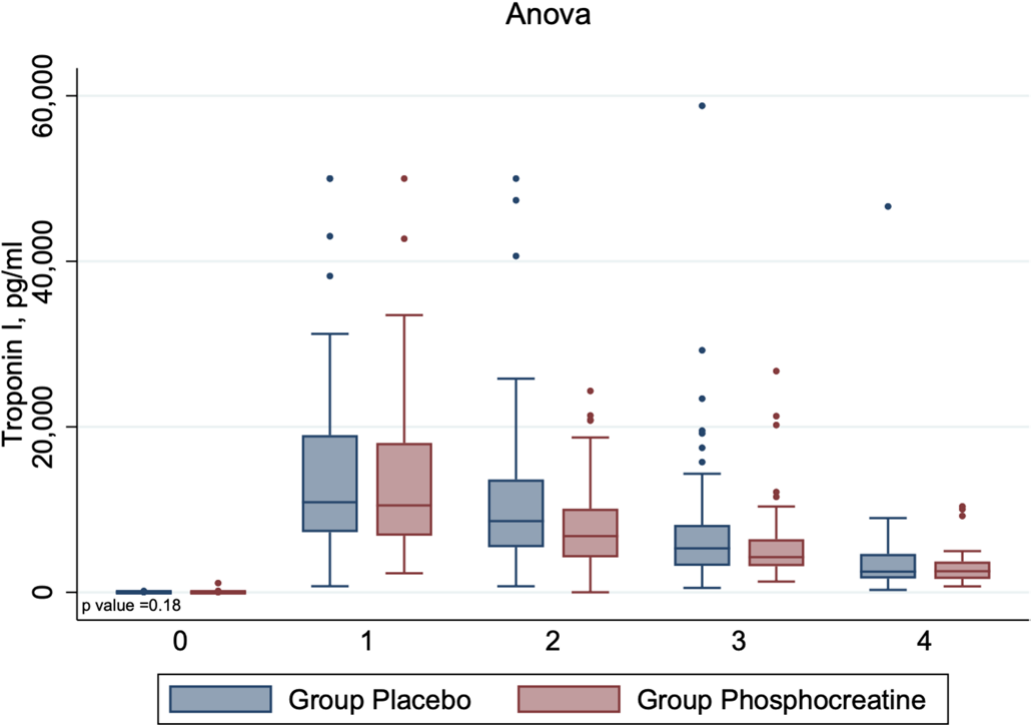
Box-plot graphic display of changes in postoperative troponin I concentration in the two study groups. Troponin I concentration was measured at baseline (0), after ICU admission (1), and on postoperative day 1 (2), postoperative day 2 (3), and postoperative day 3 (4). Data are presented as median (25^th^-75th percentiles)

There were no differences in peak troponin postoperative values (Table 3) or in any of the secondary outcomes. In particular, there were no differences in postoperative troponin I concentration at any time point (Fig. 2) or as peak values: median (interquartile) were 11,328 (7,660–22,894) in the placebo group versus 10,508 (6,838–19,034) in the PCr group (*p* = 0.24), as well as in NT-proBNP and creatinine levels after surgery (Table 3).

**Table 3:** Troponin, creatinine, NT-proBNP levels

	Placebo (*n* = 63)	Phosphocreatine (*n* = 57)	*p*-value
Troponin I, pg/ml			
Baseline	7.9 (4.7–17.7)	4.95 (3.2–9.0)	0.18
ICU admission	10,890 (7,282–18,993)	10,508 (6,838–18,045)	
POD 1	8,609 (5,460–13,634)	6,794 (4,227–10,103)	
POD 2	5,318 (3,200–8,156)	4,255 (3,165–6,416)	
POD 3	2,505 (1,675–4,640)	2,553 (1,624–3,716)	
Peak postoperative troponin, pg/ml	11,328 (7,660–22,894)	10,508 (6,838–19,034)	0.24
NT-proBNP pg/ml			
Baseline	45.2 (8–89.1)	18.5 (5.2–79)	0.86
ICU admission	47.2 (13.5–103.7)	37.4 (19.2–76.8)	
POD 1	67.8 (28.3–137)	56.8 (29.9–101)	
POD 2	104.5 (51.6–157)	61.1 (39.2–126)	
Peak post operative NT-proBNP, pg/ml	104.5 (40.7–186,5)	73.5 (38.3–164.5)	0.36
Creatinine, µmol/L			
Baseline	92 (79–102)	87 (79–96)	0.057
POD 1	95 (82–112)	90 (80–100)	
POD 2	90.5 (75–100)	84 (68–99)	
Peak postoperative serum creatinine, µmol/L	99.5 (90–117)	100 (85–117)	0.87

Data are presented as median (25^th^-75th percentiles)

*Abbreviations*: *ICU* Intensive care unit, *POD* Postoperative day

**Fig. 3:**
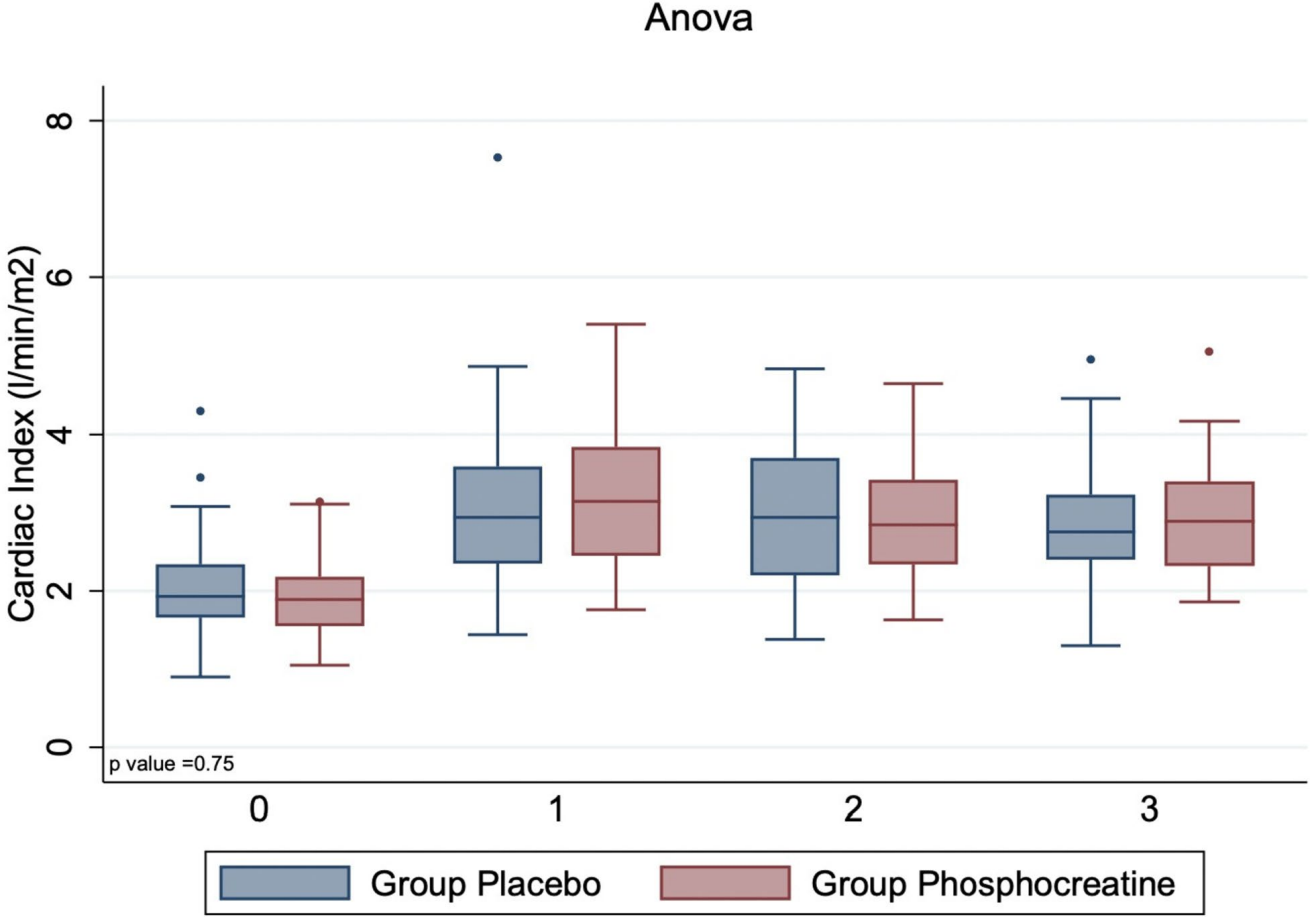
Box-plot graphic display of changes in postoperative cardiac index in the two study groups. Cardiac index was evaluated after anesthesia induction (0), 5 min after CPB (1), 6 h after Intensive Care Unit (ICU) admission (2) and on the morning of postoperative day 1 (3). Data are presented as median (25^th^-75th percentiles)

No differences were observed in cardiac index (Fig. 3), number of patients requiring inotropic support 57 (91%) vs 49 (88%) *p* = 0.60, inotropic score 4 (0–10) versus 4 (0–7) and other outcomes between the placebo and the PCr groups (Table 4). One patient in the placebo group died.

**Table 4:** Postoperative outcomes

	Placebo (*n* = 63)	Phosphocreatine (*n* = 57)	*p*-value
Number of patients requiring vasopressor/inotropic drugs, n (%)	57 (91%)	49 (88%)	0.60
LVEF on POD 1, %—median (IQR)	56 (50–62)	55 (52–59)	0.89
Inotropic score on POD 1—median (IQR)	4 (0–10)	4 (0–7)	0.47
SOFA score on POD 1 – mean ± SD	5.45 ± 2.65	5.25 ± 2.33	0.83
Mechanical ventilation, hours	6 (4–10)	6 (4–11)	0.70
New onset atrial fibrillation, n (%)	5/43 (12%)	8/30 (27%)	0.13
Duration of ICU stay, hours—median (IQR)	17 (16–20)	18 (16–20)	0.58
Length of hospitalization, days—median (IQR)	14 (11–16)	13 (11–15)	0.64
Myocardial infarction, n (%)	2 (3.2%)	2 (3.5%)	0.92
Acute kidney injury, n (%)	6 (9.5%)	9 (16%)	0.43
Neurological damage type I, n (%)	0 (0%)	1 (1.7%)	0.29
Neurological damage type II, n (%)	4 (6.3%)	5 (8.8%)	0.61
Mediastinitis, n (%)	3 (4.8%)	1 (1.7%)	0.36
Wound infection, n (%)	2 (3.2%)	2 (3.5%)	0.92
Pneumonia, n (%)	2 (3.2%)	2 (3.5%)	0.92
30-day mortality, n (%)	1 (4.3%)	0 (0%)	0.40

*Abbreviations*: *IQR* Inter Quartile Range (25^th^-75^th^ percentiles), *LVEF* Left Ventricular Ejection Fraction, *POD* Post Operative Day, *SD* Standard Deviation, *SOFA* Sequential Organ Failure Assessment, *ICU* Intensive Care Unit

## Discussion

### Key findings

In this double-blind, randomized controlled trial the authors found that PCr administration to patients undergoing double/triple valve surgery under cardiopulmonary bypass was safe but not associated with a decrease in troponin I concentration nor in an improvement in any of the numerous secondary outcomes.

### Relationship with previous studies

Endogenous PCr plays a pivotal role in normal myocardial metabolism. While experimental animals with completely undetectable levels of creatine and PCr demonstrated increased susceptibility to acute stress [9], overexpression of creatine transporter in ex vivo perfused hearts significantly reduced ischemia/reperfusion injury and improved functional recovery [10].

Cardioprotective properties of PCr were first described in early seventies by Parratt J. and Marshall R., who showed that exogenous PCr supplementation protected isolated cardiac muscle from the consequences of anoxia [11]. Moreover, the infusion of PCr into the left ventricle of dogs with acute ligation of the left coronary artery markedly reduced the number of ventricular ectopic beats [12].

Numerous studies showed that exogenous PCr administration was able to decrease intraoperative inotrope use [13–15], improve spontaneous recovery of the cardiac rhythm immediately after aortic declamping [16–19], and reduce myocardial injury [13, 20–24].

In a meta-analysis of 41 randomized controlled and case-matched studies, Landoni et al. evaluated the influence of PCr supplementation in patients with ischemic heart disease or chronic heart failure or those undergoing cardiac surgery on all-cause mortality [5]. Patients receiving PCr had lower mortality for any cause when compared with the control group (61/1731 (3.5%) vs 177/1667 (10.6%); OR: 0.71, 95% CI: 0.51–0.99; *p* = 0.04; with 3400 patients and 22 trials included). One of the major limitations of this study was that most of the included studies had an unclear or high risk of bias.

A recent meta-analysis of randomized studies evaluated whether PCr can protect the heart from ischemia and decrease the need for inotropes/vasopressors after cardiac surgery [6]. The authors analyzed 1,948 patients from 26 RCTs. In the majority of these trials PCr was added to cardioplegic solution to achieve concentration on average of 10 mmol/L. The meta-analysis showed that PCr added to cardioplegic solution or/and administered intravenously significantly reduced myocardial injury, intraoperative inotropic support, incidence of major arrhythmias and increased LVEF. PCr was also associated with increased rate of spontaneous recovery of the cardiac rhythm after removal of aortic cross-clamp. The main limitation of this meta-analysis was low methodologic quality of included studies.

Thus, beneficial effects of exogenous PCr were observed in studies with high risk of bias. Our study was a first randomized, double-blind, placebo-controlled high-quality trial and this can explain the difference in the findings.

While in the majority of previously published studies St. Thomas’ Hospital solution was used for myocardial protection, we utilized Custodiol cardioplegia which is a better method of myocardial protection in patients undergoing valvular procedures [25]. Therefore, a possible interpretation of our findings is that PCr offers no additional cardiac protection when Custodiol cardioplegia is used in valve heart surgery. One might also argue that the dose of PCr used in our study was not enough to produce sufficient myocardial protective effect. However, the dose of PCr administered to our patients (13 g of PCr perioperatively) was higher than previously reported [5] to ensure biochemical and clinical effects.

### Implications of study findings

In normal conditions, creatine is metabolized to creatinine, which is then excreted in the urine. In young healthy adults, supplementation with exogenous creatinine doesn’t impact kidney function and plasma creatinine concentration [26]. In cardiac surgical patients who are at the high risk of development of perioperative acute kidney injury, supplementation with large doses of PCr might potentially increase plasma creatinine concentrations. In our study, no between group differences were observed in the level of this metabolite. But even if administration of PCr was safe and not associated with increase in concentration of creatinine after surgery, there were no beneficial effects of this drug in terms of myocardial protection and clinical outcomes in patients after valvular surgery procedures.

### Study strengths and limitations

Our study has several strengths. Firstly, it was the first randomized, double-blind, placebo-controlled study in this area. Secondly, our cohort included high-risk cohort of patients undergoing double or triple valve surgery. Thirdly, patients were followed up for 30 days for vital status assessment.

One limitation of current study was that, due to pilot design of the study, no power calculation was performed. Another limitation of the study was that sevoflurane, which has cardioprotective properties, was used as a main anesthetic agent. This might have influenced the results of the study.

## Conclusion

In conclusion, the authors demonstrated that PCr administration to patients undergoing double/triple valve surgery under cardiopulmonary bypass is safe but not associated with a decrease in troponin I concentration. PCr had no beneficial effect on clinical outcomes after surgery.

## Data Availability

The datasets generated and/or analyzed during the current study are available from the corresponding author on reasonable request.

## Abbreviations

AKI: Acute kidney injury
ATP: Adenosine triphosphate
CPB: Cardiopulmonary bypass
ICU: Intensive Care Unit
IQR: Interquartile ranges
KDIGO: Kidney Disease: Improving Global Outcomes
LVEF: Left Ventricular Ejection Fraction
MAP: Mean arterial pressure
PCr: Phosphocreatine
RCT: Randomized Controlled Trial
SD: Standard Deviation
SOFA: Sequential Organ Failure Assessment
